# A survey report on the donkey original breeding farms in China: Current aspects and future prospective

**DOI:** 10.3389/fvets.2023.1126138

**Published:** 2023-03-16

**Authors:** Zhenwei Zhang, Bingjian Huang, Yonghui Wang, Mingxia Zhu, Guiqin Liu, Changfa Wang

**Affiliations:** Liaocheng Research Institute of Donkey High-Efficiency Breeding and Ecological Feeding, Agricultural Science and Engineering School, Liaocheng University, Liaocheng, China

**Keywords:** donkey farms, donkey breeds, reproductive parameters, growth performance, lactation performance

## Abstract

**Introduction:**

The number of the large-scale donkey breeding farms in China has increased dramatically. However, information regarding the situation of a Chinese donkey population under large-scale donkey breeding farms is limited.

**Methods:**

This survey report was conducted using questionnaires online to investigate the current situation of the donkey original breeding farms in China, in terms of donkey stock, local breeds, reproductive parameters, growth and lactation performance, and future perspectives. China has developed the donkey reserve system based on national, provincial and non-governmental (self-own) donkey original breeding farms.

**Results:**

In the present study, a total of 38 donkey original breeding farms concentrated in Northern of China were studied, and 52% of them keep their donkeys with a stocking density of 100–500 donkeys. China is rich in various local donkey breeds, and 16 local donkey breeds including large-sized, medium-sized and small-sized breeds were collected in our survey. Dezhou donkey are prevalent with a percentage of more than 57% of the total donkeys, while the Cullen donkeys belong to small-sized donkey breeds are scare. The reproductive efficiency and donkey productivity were different across donkey farms, indicating potential differences in management and breeding practices between different donkey original breeding farms. The artificial insemination has been performed in these donkey farms with an average proportion of 73%. Regarding the donkey productivity, the national and provincial donkey original breeding farms showed a higher birthweight and fat content in donkey milk than self-own farms. Furthermore, our results indicate that the donkey breeds with different body size also have important influence on the reproduction parameters and donkey productivity, with the large-sized donkeys had better performance compared to the small-sized donkeys.

**Discussion:**

In summary, our survey provided valuable baseline information on the situation of donkey population dynamics in the donkey original breeding farms. However, further study is required in the future to investigate the factors such as donkey health care, management and nutrition during breeding, fattening and lactation that influence donkey productivity under large-scale farm systems.

## 1. Introduction

Domesticated donkeys (*Equus asinus*, odd–hoofed order), as a species originated from wild asses, have played an important role in human mobility and in trading activities across the Old World (1). It is a broad consensus that the donkey domestication started in Africa (1, 2). Between 5,000 and 7,000 years ago, the response of pastoralists in Northeastern Africa to the Sahara desertification prompted the domestication of donkeys (3). Then, donkeys were mainly used as draft animals and have been introduced into Europe, America, and Asia by travelers over the centuries (4).

China is one of the largest donkey breeders worldwide with a long history of nearly 4,000 years (5). In China, before the reform and open policy, donkeys were extensively raised by the smallholders traditionally work on coarse cereals farming, and they were principally employed as tractor force and transport of people and goods in agriculture (6). Therefore, donkeys are common domestic livestock housed and used in the rural areas. They played an essential role in the socioeconomic and cultural development for millions of households (4, 7). In recent years, with the rapid development of agricultural modernization and the depopulation of rural districts, donkeys have suffered a substantial decrease in their population size (8). During the past 10 years, the total number of donkeys in China has sharply declined from 4.85 to 1.97 million (Supplementary Figure S1), however, it has reached a plateau and has remained relatively stable.

Nowadays, the national diet structure in China has undergone significant changes with the increasing improvement of people's living standard (4). In addition to being content to consume pig, beef, sheep, chicken and other meat products, people are also particularly interested in traditional donkey meat and its by-products (9). The use of donkeys is changing from draft to meat, milk, hide, medicine, cosmetics and functional or other bioproducts (9). Consequently, even though it is still in the burgeoning phase, the donkey industry is an important part of animal husbandry in China. The rise and fall of the donkey industry is not only correlated with the livelihood of farmers who live in marginal areas, but also related to the development of a livestock industry and biodiversity (10).

The number of donkeys in China is mainly concentrated in Liaoning, Shanxi, Xinjiang, Inner Mongolia, Gansu and Shandong Province (4). In China, the donkey industry is based on local breeds and crossbreeds raised under smallholder, semi-intensive and intensive systems. There are nearly 30 donkey breeds in China, which are rich in donkey resources (4). Due to their long gestation period, donkeys are relatively expensive to breed for farmers. Recently, it is reported that a series of favorable polices have adopted by Chinese Government to provide subsidies to encourage donkey breeding (11). Therefore, the number of large-scale donkey breeding farms in China has dramatically increased in recent years. According to the China Statistical Yearbook (12), the number of donkeys raised on large-scale donkey breeding farms accounts for more than 13% of the total donkey population, and the majority of these donkey breeding farms are located in North, Northwest and Northeast China. These donkey farming systems are unlike any seen before globally in terms of their scale, intensity, technology and level of investment. In addition, they are crucial for the preservation of donkey genetic resources as well as to satisfy the demand for the more diversified applications of donkey products (11).

However, to the best of our knowledge, rare information is available regarding the population dynamics of Chinese donkeys under large-scale donkey breeding farms. Hence, the present survey report was conducted to ascertain the current aspects of the large-scale donkey breeding farms in China, including the donkey stock, breeds, donkey production performance, and future prospective to the development of the donkey industry.

## 2. Materials and methods

### 2.1. Study area

**Figure 1:**
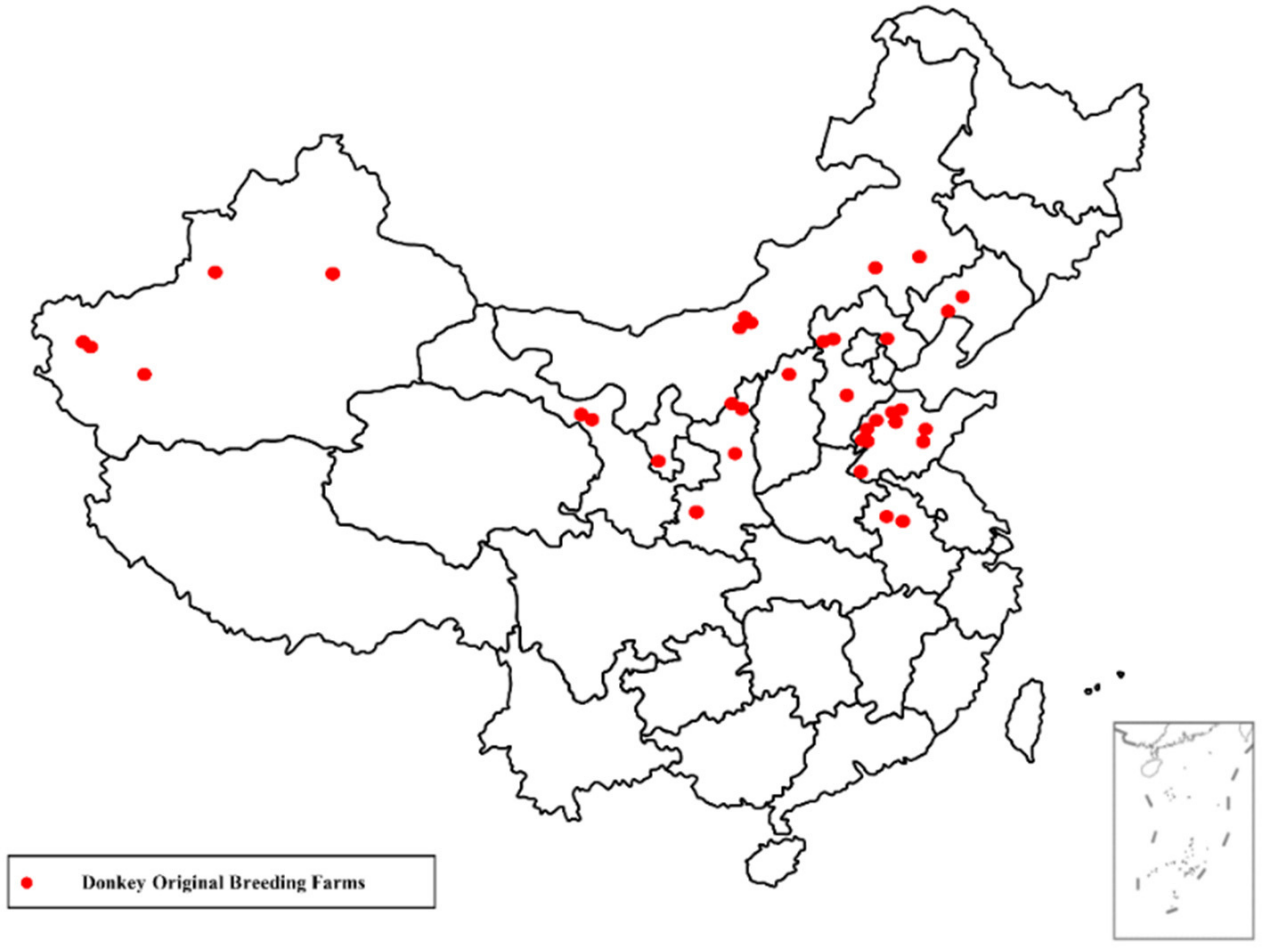
Map showing the approximate location of the participants completed the questionnaire in Northern region of China.

The present survey was conducted from September to October 2022 in the Northern Region of China, included Xinjiang (73°40′-96°23′E and 34°22′-49°10′N), Gansu, Inner Mongolia, Ningxia, Shanxi, Shaanxi, Hebei, Liaoning, Shandong and the North areas of Anhui Province (Figure 1), with a total area of 2.0 million square kilometers. This part of China mainly included the Northeast Plain, the North China Plain, the Loess Plateau, as well as the Inner Mongolia Plateau. Northern China is a massive region with tremendous variations in weather and climate. It's difficult to make generalizations about such a large area, the climate is mostly continental, with dry and freezing winters and summers that are warm with lots of rain. The annual precipitation is below 800 mm. During the winter months, the average temperature is below 0°C, while the summer is monsoon season with a temperature above 20°C. The region is typically very sunny with an average of 2,700 h of sunshine each year.

The donkey herds in the present study area consisted of ~1.6 million indigenous donkey breeds, which account for two-thirds of donkey populations in China. Among them, more than 30% donkeys were raised in the donkey original breeding farms. These large-scale donkey breeding farms employed the trained donkey breeders, milkers and breeding staffs. In addition, the modern donkey farms consist of sheltered stables with pasture fields, nutritional feed supplements and free access to water. These farms provide facilities and management conditions for improving welfare of donkeys such as proper ventilation, lighting and bedding materials. In these large-scale farms, artificial insemination has gained popularity, but in most donkey farms breeding by in-hand natural mating is the predominant breeding system.

### 2.2. Survey methodology and data collection

The selected farms were invited by an online questionnaire in the WeChat, and they were firstly informed to the information on the aim of the research and a brief outline of the survey. Participants were completely voluntary, anonymous and unremunerated. By clicking on an online link provided in the WeChat, respondents were directed to a survey software program (Jinshan Survey Form) where the survey started. Due to the national privacy regulations, the contact information of farms was unavailable to the researchers. If participants were interested in the findings of the present study and the follow-up in-depth interviews, they could provide the email and telephone number. The informed consent was given to the participants when they would like to provide their email address. The confidentiality of the contact information was guaranteed. This online survey program was accessible between September 20 and October 31, 2022.

The present survey included a section on farm basic information, a section on donkey reproduction, a section on donkey growth performance and a section on the lactation performance of dairy donkeys. A complete list of the questions in this survey are shown in Supplementary Table S1. The farm basic information mainly included the location, classification (national, provincial, and self-own), donkey breeds, number of donkey herds and education level of managers. The parameters related donkey reproduction were included the numbers of breeding Jacks and Jennies, age of first semen collection (year), sperm production (ml), sperm motility (%), age at first foaling (month), foaling interval (day), proportion of foals born alive (%), weaning age (month) and proportion of artificial insemination (%). With respect to details of donkey growth performance, the body weight, body height, body length, thoracic girth and the cannon bone girth of donkeys were recorded and collected. The information of lactation performance for dairy donkeys in different parity were the production results including milk yield per day (kg) and percentage fat and protein (g/100 ml).

### 2.3. Statistical analysis

The baseline survey data for each farm was filled in the questionnaire and saved as an individual file. In total, 38 farm files were compiled and merged into a single Microsoft Excel database. The database was then initially checked for correcting spelling errors, homogenizing character variables, ensuring the missing values were represented by NAs, checking for possible mistakes due to incorrect translation, forgotten decimal points, and wrong units, etc. For practical reasons and to conform to the statistical models, levels within some dependent variables from the current survey were grouped: farm classification was grouped to (1) national, (2) provincial and (3) self-own donkey original breeding farms. The age of donkeys were grouped to (1) 3-month-old, (2) 6-month-old, (3) 12-month-old, (4) 18-month-old and (5) 24-month-old. Donkey sex were divided into (1) male and (2) female groups. Donkey breeds were classified into 16 groups according to their breed.

After database cleaning, the SAS 9.4 (SAS Institute Inc., Cary, NC, USA) were applied for all descriptive and statistical analyses. Descriptive statistics that include frequencies, means, percentages, and standard deviations (SD) of variables were generated. Values are showed as means or means ± SD. Significance between means was estimated by one-way analysis of variance followed by a multiple comparisons test (Tukey/Kramer). Differences were declared statistically significant when *P* < 0.05. Correlations between donkey body weight and body measurements were analyzed using PROC CORR procedure in SAS 9.4. All figures were conducted using the GraphPad Prism 8.0 version.

## 3. Results

### 3.1. Basic information of donkey farms

**Figure 2:**
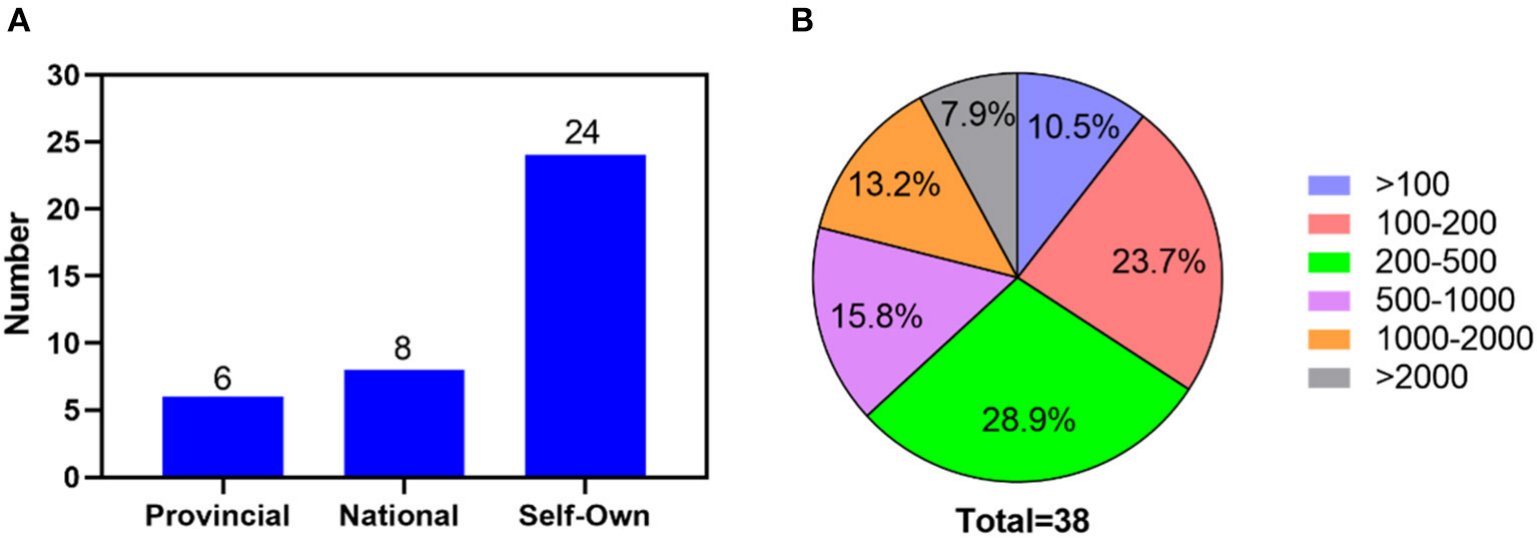
The donkey farm classification **(A)** and the proportion of donkey farms **(B)** with different amount on hand.

A total of 38 donkey farms were investigated in the present survey study, which included 6 provincial, 8 national and 24 self-own donkey original breeding farms (Figure 2A). Among them, almost 29% had a stocking density of 200–500 donkeys/farm, more than 23% of the farms surveyed had a livestock stock of 100–200 donkeys per farm (Figure 2B). Less than 8% of farms have more than 2,000 donkeys on hand, which is a very small percentage. The core breeding donkey group in large-scale donkey farms in China is usually comprised of Jacks and Jennies. The number of Jacks is always limited (< 30), but the number of Jennies is mostly between 100 and 300 in the donkey original breeding farms (Supplementary Figure S2). In total, 24,957 donkeys were investigated in the present study. It included 1,029 Jacks, 17,262 Jennies and 6,666 feedlotting donkeys.

**Figure 3:**
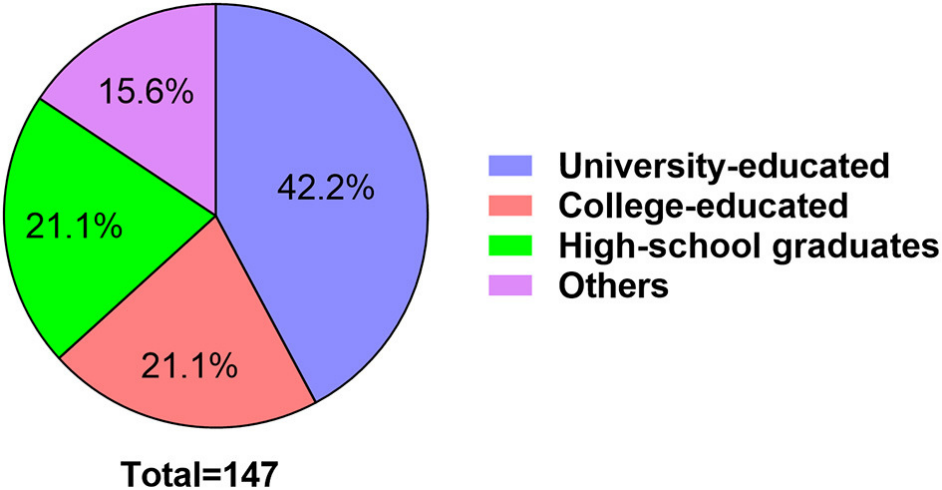
Education level of donkey farm managers.

The education level of donkey farm managers was analyzed (Figure 3), more than half (42.2% university educated and 21.1% college educated) of the farm managers had a college degree in this study.

**Figure 4:**
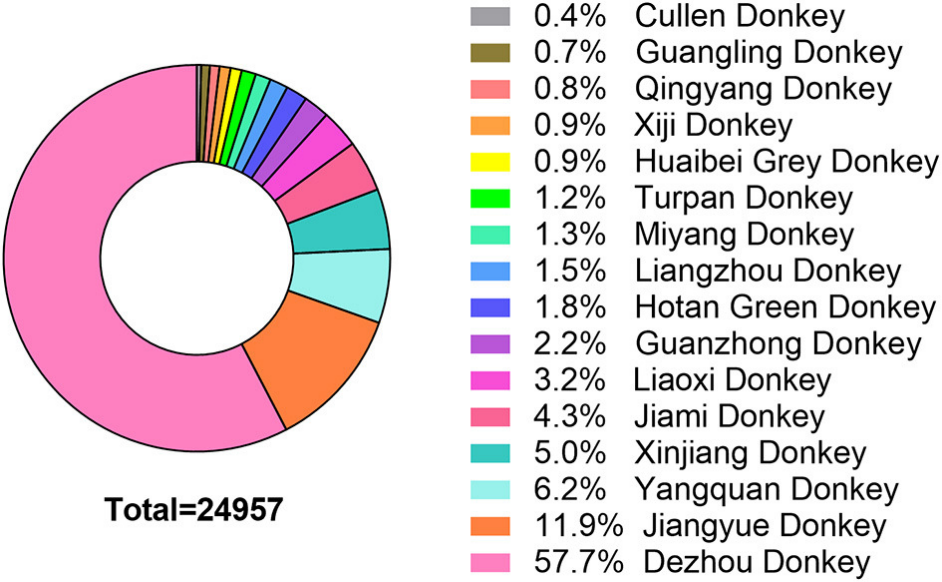
Donkey breeds and their proportions in the present survey.

In the present study, there were 16 local donkey breeds were surveyed (Figure 4). Among them, the top 5 predominantly local breeds were Dezhou donkey (57.7%), Jiangyue donkey (11.9%), Yangquan donkey (6.2%), Xinjiang donkey (5.0%) and Jiami donkey (4.3%). The proportion of Cullen donkey, Guangling donkey, Qingyang donkey, Xiji donkey and Huaibei Grey donkey was less 1%.

### 3.2. Reproductive parameters

The reproduction parameters of Jacks in different donkey original breeding farms were analyzed (Table 1). The overall mean age of first semen collection was 3.1 years (*n* = 619), the overall mean sperm production of Jacks per day was 44.2 ml/d, and the overall mean of sperm motility was 82.5%. There was no obvious difference for age of first semen collection and sperm motility between the provincial, national and self-own donkey original breeding farms. But Jacks in the provincial and national donkey original breeding farms had higher sperm production (75.0 and 50.3 ml/d) compared with Jacks in the self-own donkey original breeding farms (39.0 mL/d).

**Table 1:** Reproduction parameters of Jennies in different donkey original breeding farms in China.

**Items**	**Provincial**	**National**	**Self-own**	**Average**
**Jack**				
Numbers of breeding Jacks (*n*)	48	63	508	619
Age of first semen collection (year)	3.3 ± 0.9	3.3 ± 0.3	3.0 ± 0.3	3.1 ± 0.2
Sperm production (ml/d)	75.0 ± 0.0	50.3 ± 11.9	39.0 ± 9.8	44.2 ± 7.4
Sperm motility (%)	80.0 ± 4.2	83.3 ± 2.7	82.5 ± 4.0	82.5 ± 2.1
**Jenny**				
Number of breeding Jennies (*n*)	1,004	1,098	8,057	10,159
Age at first foaling (month)	39.0 ± 1.7	38.5 ± 1.3	39.7 ± 2.2	39.4 ± 1.5
Foaling interval (day)	400.0 ± 18.1	453.7 ± 27.6	400.2 ± 7.4	410.9 ± 8.4
Foaling alive rate (%)	87.8 ± 3.4	80.5 ± 4.2	83.3 ± 1.8	83.3 ± 1.5
Weaning age (month)	5.5 ± 0.5	5.0 ± 0.7	7.2 ± 0.7	6.5 ± 0.5
Proportion of artificial insemination (%)	42.5 ± 7.5	71.0 ± 12.1	81.1 ± 6.7	73.1 ± 6.0

The overall mean age of first foaling of Jennies was 39.4 months (Table 1, *n* = 10,159); the overall mean foaling interval of Jennies was 410.9 days; the overall mean of foaling alive rate was 83.3%; and the overall mean weaning age of foals is 6.5 months. Donkey foals in the provincial and national donkey original breeding farms had lower weaning age (5.5 and 5.0 months) than foals in the self-own donkey original breeding farms (7.2 months). In addition, the foaling intervals of Jennies in the national donkey original breeding farms (453 days) was higher than the donkeys in the provincial (400.0 days) and self-own (400.2 days) donkey original breeding farms. The proportion of artificial insemination in donkey original breeding farms was ranked as provincial > national > self-own. There was no remarkable difference for the age of first foaling and the foaling alive rate among the provincial, national and self-own donkey original breeding farms.

Table 2 showed the reproduction parameters of Jacks within different donkey breeds. The reproduction parameters of Cullen donkey, Qingyang donkey, Xiji donkey, Huaibei Grey donkey, Turpan donkey, Liangzhou donkey, Hotan Green donkey and Liaoxi donkey were not collected. The age of first semen collection for Jiangyue donkey (4.5 years) and Guangling donkey (4.0 year) was remarkable longer than Jiami donkey (1.9 years) and Miyang donkey (2.0 years). The top 3 predominantly sperm production was Jiami donkey, Miyang donkey and Dezhou donkey, but the sperm production of Jiangyue donkey, Xinjiang donkey and Yangquan donkey was < 10 ml. The sperm motility for local donkey breeds in China were ranged from 74.0% to 87.0%.

**Table 2:** Reproduction parameters of Jacks within different donkey breeds in China.

**Donkey breeds**	**Numbers of Jacks (*n*)**	**Age of first semen collection (year)**	**Sperm production (ml/d)**	**Sperm motility (%)**
Guangling donkey	2	4.0	37.5	87.0
Miyang donkey	78	2.0	75.0	–
Guanzhong donkey	176	2.9 ± 0.6	26.7 ± 19.2	75.0
Jiami donkey	72	1.9	76.0	–
Xinjiang donkey	12	2.5	8.0	70.0
Yangquan donkey	7	3.0 ± 1.0	8.0	85.0
Jiangyue donkey	20	4.5 ± 1.5	4.5	74.0
Dezhou donkey	272	3.0 ± 0.3	51.7 ± 10.7	83.9 ± 2.4

–, not collected.

Table 3 displayed the reproduction parameters of Jennies in different donkey breeds. The reproduction parameters of Cullen donkey, Qingyang donkey and Liaoxi donkey were not collected. Except Jiangyue donkey (72 months), the age at first foaling among local donkey breeds were mainly ranged from 33.0 to 48 months. Apart from Hotan Green donkeys have a long gestation period of 480 days, the foaling interval of other donkey breeds were primarily between 360.0 and 428.3 days. The foaling alive rate was high for the local donkey breeds in China, except Guanzhong donkeys (73.3%), most of them are higher than 80%. The weaning age among different donkey breeds fluctuates greatly. The weaning age of Xiji donkeys (12.0 months), Miyang donkeys (10.8 months), Liangzhou donkeys (10.5 months), Jiangyue donkeys (10.0 months) and Jiami donkeys (9.6 months) were obvious longer compared with Guangling donkeys (4.0 months), Huaibei Grey donkeys (4.5 months) and Dezhou donkeys (5.2 months). The artificial insemination in donkey breeding has only been applied in Hotan Green donkeys, Guanzhong donkeys, Xinjiang donkeys, Yangquan donkeys and Dezhou donkeys, and the proportion of artificial insemination is low (30–100%).

**Table 3:** Reproduction parameters of Jennies in different donkey breeds in China.

**Breed**	**Number of Jennies (*n*)**	**Age at first foaling (month)**	**Foaling Interval (day)**	**Proportion of foals born alive (%)**	**Weaning age (month)**	**Proportion of artificial insemination (%)**
Guangling donkey	95	42.0	420.0	90.0	4.0	–
Xiji donkey	60	36.0	360.0	90.0	12.0	–
Huaibei grey donkey	120	36.0	417.5 ± 32.5	80.0	4.5 ± 1.5	–
Turpan donkey	100	42.0	385.0	85.0	6.0	–
Miyang donkey	230	36.0	380.0	86.0	10.8	–
Liangzhou donkey	140	33.0 ± 3.0	406.5 ± 48.5	90.0	10.5 ± 1.5	–
Hotan Green donkey	168	38.0	480.0	90.0	6.0	50
Guanzhong donkey	615	36.0	428.3 ± 46.9	73.3 ± 4.4	8.4 ± 1.2	50–85
Jiami donkey	300	36.0	390.0	90	9.6	–
Xinjiang donkey	630	48.0	420.0	90.0	6.0	85
Yangquan donkey	1,255	39.0 ± 9.0	382.5 ± 17.5	88.0 ± 2.0	6.5 ± 0.5	70–100
Jiangyue donkey	480	72.0	391.0	80.0	10.0	–
Dezhou donkey	5,966	38.9 ± 1.9	414.3 ± 16.1	82.1 ± 2.8	5.2 ± 0.4	30–100

### 3.3. Growth performances

As shown in Table 4, the body weight of donkeys among different donkey original breeding farms in China were compared. Regardless male and female donkeys, the birthweight of donkeys was ranked as national (>31.5 kg) >provincial (>23.9 kg)> self-own (>27.1 kg) donkey original breeding farms. In addition, with age increasing, the body weight of donkeys in national and provincial donkey original breeding farms was still higher than self-own donkey original breeding farms.

**Table 4:** The body weight of donkeys among different donkey original breeding farms in China.

**Items**	**Age (months)**	**Body weight (kg)**	
		**Male**	**Female**
Provincial	0	29.1 ± 4.5	23.9 ± 2.1
	3	–	–
	6	110.0	112.0
	12	157.5 ± 7.5	160.0
	18	–	–
	24	254.0	248.0
National	0	32.8 ± 1.9	31.5 ± 2.5
	3	–	–
	6	114.6 ± 13.6	131.8 ± 2.2
	12	161.1 ± 23.1	182.5 ± 11.5
	18	223.8 ± 31.2	226.9 ± 8.5
	24	260.1 ± 21.5	246.3 ± 3.7
Self-own	0	27.1 ± 1.8	29.0 ± 1.8
	3	53.4 ± 7.6	51.9 ± 7.1
	6	101.3 ± 12.4	106.2 ± 21.8
	12	142.4 ± 5.6	179.2 ± 22.8
	18	201.4 ± 10.4	220.4 ± 35.4
	24	227.0 ± 29.2	250 ± 25.2

**Figure 5:**
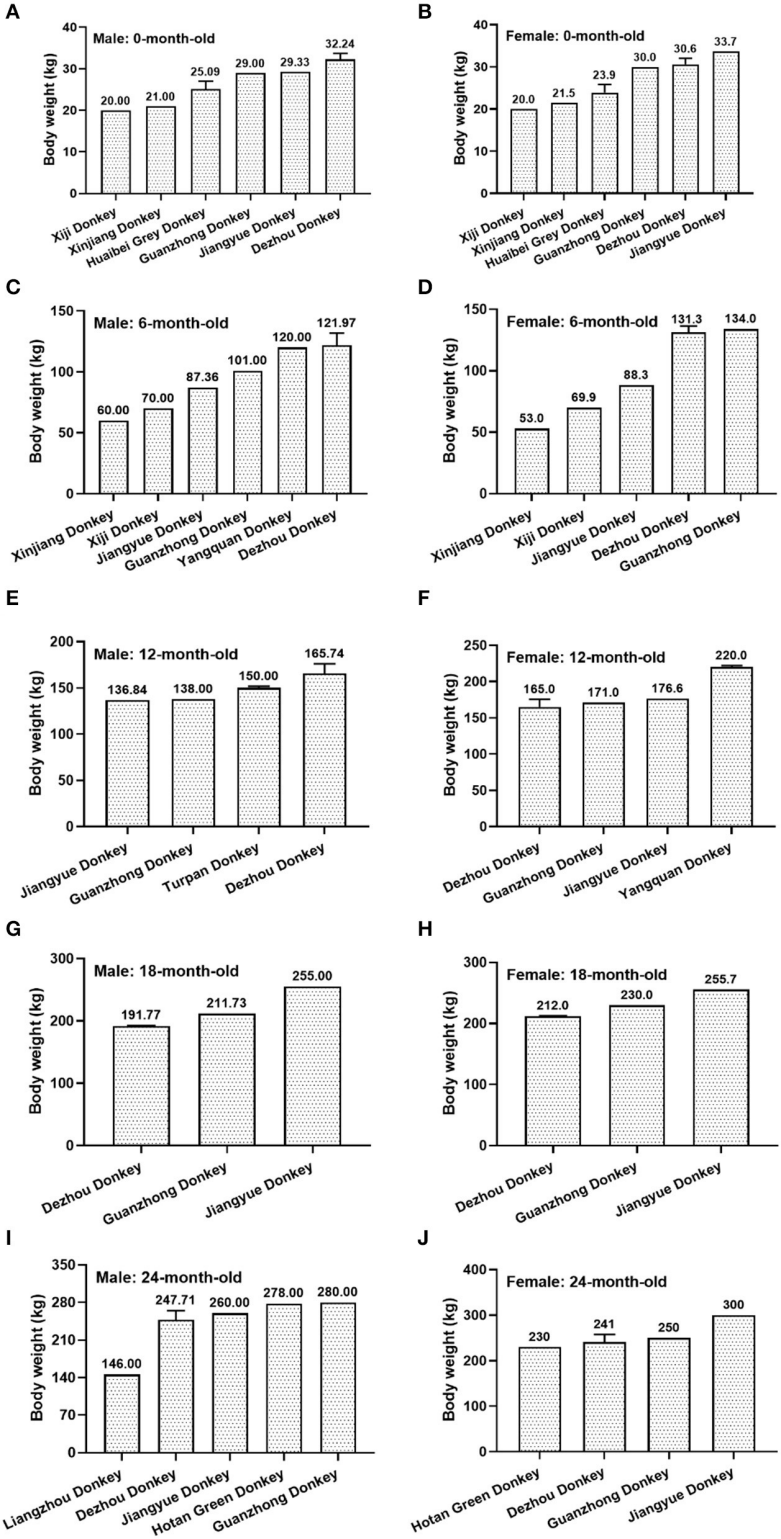
The body weight of different donkey breeds at different months of age. **(A)** 0-month-old male donkeys; **(B)** 0-month-old female donkeys; **(C)** 6-month-old male donkeys; **(D)** 6-month-old female donkeys; **(E)** 12-month-old male donkeys; **(F)** 12-month-old female donkeys; **(G)** 18-month-old male donkeys; **(H)** 18-month-old female donkeys; **(I)** 24-month-old male donkeys; **(J)** 24-month-old female donkeys.

Regardless male and female donkeys, the birthweight of Xiji donkeys (20.0 kg) and Xinjiang donkeys (>21.0 kg) is lower than Guanzhong donkeys (>29.0 kg), Jiangyue donkeys (>29.0 kg) and Dezhou donkeys (>32.0 kg, Figure 5). The body weight of donkeys at 6-month-old were ranked as Xinjiang donkey < Xiji donkey < Jiangyue donkey < Guanzhong donkey. There was still difference among different donkey breeds when they are more than 12-month-old.

**Figure 6:**
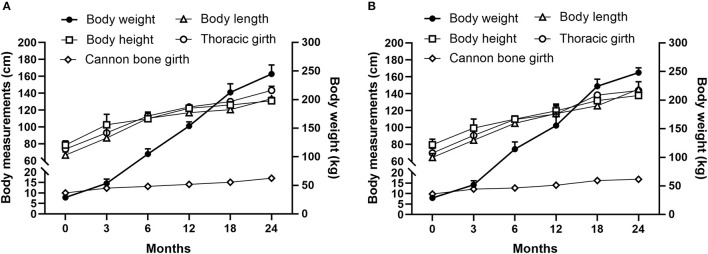
The body weight and body measurements of male [**(A)**, *n* = 104] and female [**(B)**, *n* = 100] donkey foals at different months of age.

Both the body weight and body measurements of donkey foals gradually increased with age (Figure 6). In addition, with the body weight increasing, the body measurements (including body height, body length, thoracic girth and cannon bone girth) increased gradually.

As shown in Table 5, the body weight and body measurements of donkeys, including body height, body length, thoracic girth and cannon bone girth, had positive correlations for both male and female donkeys (Table 5, R^2^ value ranged from 0.86 to 0.98, *P* < 0.05).

**Table 5:** The correlations between body weight and body measurements of donkeys.

**Items**	**Sex**	**Body weight**	**Body height**	**Body length**	**Thoracic girth**
Body height	Male	0.86^*^			
	Female	0.92^*^			
Body length	Male	0.86^*^	0.92^*^		
	Female	0.90^*^	0.92^*^		
Thoracic girth	Male	0.90^*^	0.93^*^	0.98^*^	
	Female	0.94^*^	0.96^*^	0.95^*^	
Cannon bone girth	Male	0.89^*^	0.92^*^	0.92^*^	0.93^*^
	Female	0.91^*^	0.93^*^	0.87^*^	0.95^*^

^*^P < 0.05.

### 3.4. Lactation performance

The lactation production of dairy donkeys in different donkey original breeding farms were analyzed (Table 6). The overall mean milk yield of dairy donkeys was 2.6 kg, the overall mean of Somatic Cell Count (SCC) was 18.8 × 10^3^ cells/ml, and the overall mean of fat and protein content was 0.7 g/100 ml and 2.0 g/100 ml, respectively. No obvious difference occurred for donkey milk yield between different donkey original breeding farms. But the milk protein rate among different farms ranked as national (2.4 g/100 ml) > provincial (2.0 g/100 ml) >self-own (1.8 g/100 ml).

**Table 6:** Lactation production of dairy donkeys in different donkey original breeding farms in China.

**Lactation production**	**Provincial**	**National**	**Self-own**	**Average**
Milk yield (kg)	3.1 ± 1.0	2.4 ± 0.3	2.6 ± 0.3	2.6 ± 0.2
Fat content (g/100 ml)	0.8 ± 0.3	0.4	0.8 ± 0.1	0.7 ± 0.1
Protein content (g/100 ml)	2.0	2.4 ± 0.7	1.8 ± 0.1	2.0 ± 0.2
SCC ( × 10^3^ cells/ml)	27.0	25.0	16.4 ± 6.7	18.8 ± 5.2

SCC, somatic cell count.

The lactation production of dairy donkeys within different donkey breeds were also compared (Table 7). There was obvious difference for milk yield among local donkey breeds, and the milk yield ranked as Yangquan donkey (3.1 kg) > Dehou donkey (2.9 kg) > Jiangyue donkey (2.3 kg) > Hotan Green donkey (2.1 kg) > Turpan donkey (1.6 kg) > Xinjiang donkey (1.5 kg). There was no significant difference for milk fat rate and milk protein rate among local donkey breeds, but the SCC of Dezhou donkeys (34.0 × 10^3^ cells/ml) was remarkable higher compared with others (< 10.0 × 10^3^ cells/ml).

**Table 7:** Lactation production of dairy donkeys within different donkey breeds in China.

**Breed**	**Numbers (n)**	**Milk yield (kg)**	**Fat content (g/100 ml)**	**Protein content (g/100 ml)**	**SCC ( × 10^3^ cells/ml)**
Turpan donkey	20	1.6			
Hotan green donkey	22	2.1	0.6	2.0	8.5
Xinjiang donkey	200	1.5			
Yangquan donkey	689	3.1 ± 1.9	0.3	2.0	10.0
Jiangyue donkey	1,000	2.3 ± 0.3	0.7 ± 0.1	1.6 ± 0.0	10.0 ± 0.0
Dezhou donkey	2,516	2.9 ± 0.3	0.7 ± 0.1	2.1 ± 0.3	34.0 ± 8.0

SCC, somatic cell count.

**Figure 7:**
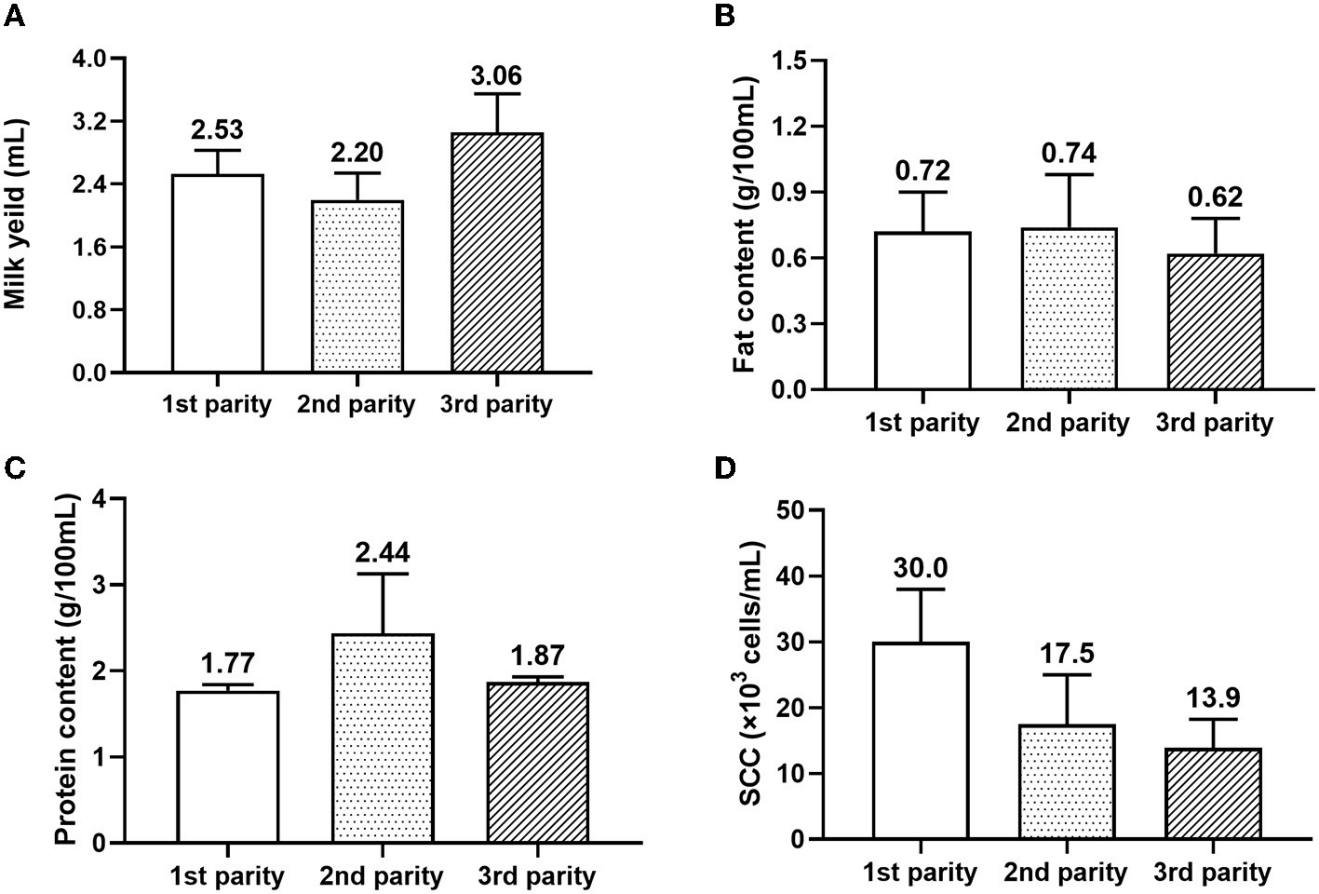
Lactation production of dairy donkeys within different parity. SCC, somatic cell count. **(A)** Milk yeild; **(B)** Fat content; **(C)** Protein content; **(D)** SCC.

Figure 7 displayed the lactation production of dairy donkeys within different parity. The milk yield of dairy donkeys in 3rd parity was higher than donkeys in 1st and 2nd parity. But there was no obvious difference for milk fat rate and milk protein rate among different parities. In contrast, with the parity increased, the SCC of dairy donkeys gradually decreased.

## 4. Discussion

### 4.1. Basic information of donkey farms in China

Donkey husbandry in China is closely related to a cultural, economic, social and ecological heritage, and it is previously importance in human feeding, hybrids breeding, and transportation of cargo and people (4, 13). Although the donkey population in China has declined over the last decade owing to the mechanization in transport and agriculture, the donkey farming for meat, milk and hide production has become an important industry in recent years (14, 15).

In the previous study, a cross-sectional survey was conducted by Deng et al. (16) in 40 villages for smallholder farms regarding demographics, health care, and management factors of donkeys in Northeastern China. However, essential information on donkey population dynamics of the original breeding farms is scarce. The objective of the present manuscript was to investigate the current situation of the donkey original breeding farms in China, in terms of donkey stock, local breeds, growth and lactation performance, reproductive parameters, and principal uses and perspectives. Our comprehensive survey should be useful in providing scientific reference information for subsequent studies as well as improving the performance and welfare of donkeys in China.

The farm sample for the survey study was selected using a stratified random sampling method by online questionnaire in the WeChat and participants on a voluntary basis. This is in agreement with the previous surveys using online questionnaires related to donkeys (17). In the current study, the participation bias cannot be excluded as it is possible that the subject of the survey may have attracted more caring and forthright donkey managers, but the online method with WeChat platform may have refrained some managers from responding. More than 90% participants we invited were glad to take part in the survey; therefore, the farm sample could be representative of the population.

In the last 20 years, the structure of animal husbandry in China has changed dramatically as cattle feeding and pig and poultry production have transitioned to fewer but larger operations (18, 19). The transformation has been increased productivity and benefited sustainability, producers and consumers. In contrast, the donkey industry is in the burgeoning phase in China (4). Over 70% of donkeys are raised under extensive conditions on smallholder farms (16). However, during the past 10 years, the donkey industry has undergone substantial changes. A large number of donkey original breeding farms have been built in Shandong, Inner Mongolia, Xinjiang, Gansu, Ningxia, Hebei, Shaanxi and Shanxi Provinces, shifting from traditional extensive farming to semi-intensive and intensive farming (4). Currently, the number of donkeys raised on large-scale farms accounts for 13% of the total donkey population (11). These donkey farms play an important role in the genetic conservation and germplasm resource utilization of local donkey breeds.

In order to avoid further loss of the important genetic material of donkey breeds, the Ministry of Agriculture and Rural Affairs of the People's Republic China decided to include conservation of animal genetic resources among its priorities. The primary reason was concern about genetic diversity loss caused by the extinction of some donkey breeds and populations. Because livestock progress and future development are linked to genetic variability, such losses may limit options for animal improvement research (20). Recently, both governments and non-governmental organizations are involved in the conservation and the innovative development of donkey genetic resources in China. Nowadays, China has developed a donkey reserve system based around national, provincial and non-governmental donkey original breeding farms. In the present study, a total of 38 donkey farms were investigated, which included 6 provincial, 8 national and 24 self-own donkey original breeding farms. In China, the provincial donkey original breeding farms are authorized by Department of Agriculture and Rural Affairs of Province; the national donkey original breeding farms are authorized by Ministry of Agriculture and Rural Affairs of the People's Republic China; and other farms were constructed voluntarily with the policy support of local government or organizations. These farms were mainly concentrated in Northern region of China that covered the majority of large-scale donkey farms. Information given by the respondents revealed that 52% of them keep their donkeys with a stocking density of 100–500 donkeys, while only 3 donkey farms keep donkeys more 2,000. The core breeding donkey group in the donkey original breeding farms in China is generally comprised of Jacks and Jennies, which contains at least 5 families, more than 10 Jacks, and more than 100 Jennies. In terms of educational level, most managers of donkey original breeding farms had completed college education. Therefore, the formal education may have improved the management and health care of the donkeys compared to smallholder farm owners (16).

### 4.2. Local donkey breeds in China

China hosts various local donkey breeds, which primarily distributed in the dry, semiarid, arid, and warm climates of Northern China around the Huanghe River Basin. There are ~24 registered indigenous donkey breeds as listed in Animal Genetic Resources in China: Horses, Donkeys, Camels (2011). These donkey breeds vary in body weights and sizes ranging from 130 to 260 kg and body height measuring from 110 cm to 130 cm. Based on morphological characteristics, the donkeys can be divided into large-sized (Guanzhong donkeys, Jiangyue donkeys, and Dezhou donkeys), medium-sized (Jiami donkeys, Miyang donkeys, and Qingyang donkeys) and small-sized (Kulun donkeys, Xinjiang donkeys, and Yunnan donkeys) breeds, with heights above 130 cm, between 115 cm and 125 cm, and below 110 cm, respectively (4). In China, a donkey original breeding farm typically raised just one local donkey breed. In the present survey, 16 local donkey breeds were collected, and Dezhou donkey are currently prevalent in China with a percentage of more than 57% of the total donkeys. This was consistent with the previous study reported by Seyiti and Kelimu (4). In contrast, the small-sized breeds, such as Cullen donkeys, Qingyang donkeys, Xiji donkeys and Huaibei Grey donkeys had a small proportion. This phenomenon could be attributed to the inferior production of meat and hide of the small-sized breeds. In the past several years, these donkeys have been slaughtered indiscriminately and may face the risk of extinction (21). Therefore, the establishment of donkey original breeding farms is crucial to the protection of genetic resources of small-sized donkey breeds.

### 4.3. Reproductive parameters of donkeys in the original breeding farms

Reproduction and breeding are important to maintain the donkey industry in China (22). The conventional breeding is currently applied in donkey original breeding farms. There is particular interest in using donkey Jacks for donkey foal production *via* both in-hand natural mating and artificial insemination (23). Recently, Deng et al. (22) investigated the foaling related parameters of Jennies in Northeast China under smallholder farm conditions. However, little information is available on the foaling related parameters of Jennies and the reproductive aspects of Jacks under large-scale farms. In China, breeding by artificial insemination has gained popularity, hence, semen collection from the Jacks is usually firstly performed in the donkey original breeding farms. The Jacks can be trained to collect with a jump-jenny in estrus, and the overall mean age of first semen collection was 3.1 years. Compared with the Jacks in the self-own donkey original breeding farms (39.0 ml/day), Jacks in the provincial and national donkey original breeding farms had higher sperm production (75.0 and 50.3 ml/day). In addition, the sperm progressive motility was detected in these farms with an overall mean of 82.5% (74.0–87.0%), which is in line with the report (70–90%) by Miragaya et al. (23). Our survey indicated that the age of semen collection of small-sized donkey breeds was younger than the large-sized donkey breeds (1.9–2.0 years old vs. 4.0–4.5 years old). In a previous study, Quartuccio et al. (24) reported a poor correlation (*P* = 0.1) between testicular volume (250–500 cm^3^) and daily sperm production in eight Ragusano Jacks (aged between 3 and 18 years), which suggested that there was no significant correlation between body size (testicular morphometric characteristics) and sperm production in donkeys. In this study, the sperm production also showed a great fluctuation among different donkey breeds regardless body size of donkeys.

The mean age at first foaling of Jennies in donkey original breeding farms was 39.4 months, earlier than 45.3 months reported in Deng et al. (22) under smallholder farm conditions. The earlier age at first service of Jennies and various management techniques between different farming systems may lead to the difference. In addition, it was also earlier compared to the 60 months reported in Ireland (25) and 57 months reported in Nigeria (1998). Furthermore, the overall mean value of foaling interval (410.9 days) was also shorter than the range (500.5 days) reported by Deng et al. (22). The shorter foaling interval of Jennies means the improvement in reproduction efficiency. However, the prolonged postpartum anestrus, silent estrus, or poor heat detection might affect the foaling interval of Jennies (22). In our study, the foaling intervals of Jennies in the national donkey original breeding farms (453 days) was longer than the donkeys in the provincial (400.0 days) and self-own (400.2 days) donkey original breeding farms. The interval of foaling to conception is an important index for determining the length of foaling interval (22). A large proportion of the Jennies bred on the first or second postpartum estrus probably shortened the foaling interval in the provincial and self-own donkey original breeding farms. The overall foaling alive rate of Jennies in the donkey original breeding farms was 83.3%, which is higher than those reported in southern Sudan (65–70%) (26). Weaning is generally one of the most essential events in the early life of donkey foals (27). Our survey also compared the weaning age of donkey foals among different donkey original breeding farms, and the provincial and national donkey original breeding farms had the lower weaning age than self-own donkey farms. The different level of management and breeding system may be the reason for explaining the differences. The overall foaling-related parameters of Jennies among different donkey breeds were surveyed in the current study. The results indicated that the reproduction characteristics, such as the age of first foaling, the foaling interval, and the foaling alive rate were not significantly distinct among local donkey breeds. But the weaning age of foals among different donkey breeds fluctuates greatly. The weaning age of the small-sized donkey foals (9.6–12 months) were longer than the large-sized donkey breeds (4.0–5.2 months). The poor body condition of small-sized donkey foals probably lead to the extension of weaning age of foals to obtain more nutrients. Although in most donkey original breeding farms breeding by in-hand natural mating is the predominant breeding system (23), artificial insemination has been carried out in these farms with an average proportion of 73.1 ± 6.0%. Artificial insemination with donkey semen makes it possible to amplify the use of good donkey sires (28, 29).

### 4.4. Growth performance of donkeys in the original breeding farms

Generally, the donkey foals naturally suckled colostrum at birth in the donkey original breeding farms in China and later milk from their dams till the weaning at the age of 6–7 months; starting from 3 months of age, they also received roughage *adlibitum* (30). Colostrum is essential for the transfer of passive immunity and health of newborn donkey foals. Information on current colostrum management practices to reduce foal morbidity and mortality is important but lacking for the donkey original breeding farms. However, the fenceline weaning for donkey foals was usually applied in China. Therefore, donkey foal stress can be minimized by modulating the degree of separation from the dam and incorporating slow changes in diet. The foal morbidity and mortality is very low in the donkey original breeding farms.

The weighing or weight estimation by using donkey body measurements are extremely useful tools for assessing the general condition and health status of donkeys when carried out on a regular basis (31). In addition, growth and development indicators are critical breeding concerns in the donkey original breeding farms of donkeys (32). However, there are very few information on various local breeds of donkeys in the research of domestic donkey growth and development. Our recent study investigated the growth characteristics of Dezhou donkeys within 0–18 months (32). In accordance with this study, a significant correlation between body weight and body measurement (such as body height, body length, thoracic girth, and cannon bone girth) was also observed in the current survey, and with the age increasing, both the body weight and body measurements of donkey foals gradually increased. The mean body height was 97.0 ± 0.60, 106.6 ± 1.27, 126.0 ± 1.03, and 129.8 ± 1.49 cm in 3-, 6-, 12- and 18-month old males and 96.3 ± 0.23, 108.8 ± 0.53, 122.8 ± 0.31, and 129.9 ± 0.38 cm in females (32). For adults, the average body height of a full-grown Dezhou donkey was 130–165 cm, and the average body length ranged from 132 to 155 cm. The body weight was 250 kg and 350 kg for adult females and males, respectively (31, 33). Apart from Dezhou donkeys, there were little records on the growth and development traits for other local breeds of donkeys. The present study may be novel in being the first study to report the growth parameters of the largest number of local breeds of donkeys in China. Many investigations have reported a high correlation coefficient between body weight and body measurement (31, 32, 34, 35). In our survey, the body weight of donkeys among different donkey original breeding farms were also compared, and the national and provincial donkey original breeding farms showed a higher growth performance than self-own farms. In practice, the breeding objectives of each original breeding farm are not exactly the same, some prefer to choose individuals with thick skin, more meat and black hair, and some prefer to choose individuals with more meat and fast growth. Therefore, the different management decisions may cause the different growth performance. Moreover, our results have also shown a statistically conclusive difference of biometric indexes (body weight and body measurements) between different donkey breeds. The body weight of small-sized donkey foals (Xiji donkeys and Xinjiang donkeys) was obviously lower compared to the medium-sized or large-sized donkeys (Guanzhong donkeys, Jiangyue donkey and Dezhou donkeys). In the selective sweep, gene annotation, functional enrichment, and differential expression analyses between large and small donkey groups, the whole-genome sequencing identified selective signals, including *NCAPG* and *LCORL*, are related to rapid growth and large body size (36). Thus, the current study provided important information on the growth and development indicators for the local breed of donkeys, which may aid in better donkey breeding (37).

### 4.5. Lactation performance of dairy donkeys in the original breeding farm

Donkey milk, which has a chemical composition very similar to breast milk, is frequently used as a treatment for allergies such as eczema and lactose intolerance in infants and young children (14, 38). In China, donkey milk is gaining prominence and has been found to have some bio-nutritional and extra-nutritional beneficial properties than cow milk (39). The overall mean milk yield of dairy donkeys within donkey original breeding farms were 2.6 kg, which is higher compared to the dairy donkey farms located in North Western Italy (40). The literature reported that fat content in donkey milk ranges from 0.3 to 1.8 g/100 ml, while the protein content is reported to be less variable, with values from 1.4 to 1.8 g/100 ml (41). This is in accordance with our results of the fat (0.7 g/100 ml) and protein content (2.0 g/100 ml) in donkey milk. However, the protein content between different donkey original breeding donkey farms was not identically. It ranked as National (2.4 g/100 ml) >provincial (2.0 g/100 ml) >self-own (1.8 g/100 ml). Values for average SCC (18.8 × 10^3^/ml) are in the range reported on previous studies on Italian and Ragusano breeds (42, 43). Furthermore, different donkey breeds in the current survey have marked differences on milk production. The milk production ranked as Yangquan donkey> Dehou donkey >Jiangyue donkey >Hotan Green donkey >Turpan donkey >Xinjiang donkey. It appears that the donkey size affected the milk production as large-sized donkeys had better performance for milk yield compared to the small-sized donkeys. But neither the fat content nor the protein content was affected by donkey breeds. Previous study has related the weight to milk production in dairy cattle (44). Milk yield increased with cows got heavier (45). Therefore, the large-sized donkeys with higher live weight than the small-sized donkeys may yield more milk. However, with the metabolic live weight of donkeys increased more than the threshold, the milk production of dairy donkey may decline as they need more nutrients for maintain metabolism. Further research is needed in the future to focus on how metabolic live weight can affect milk production in dairy donkeys.

With the donkey parity increasing, the SCC of donkey milk gradually decreased. Until now, research on the influence of parity on the SCC in dairy donkeys is scare. However, previous studies on the dairy cows had reported that the parity was associated with variation in SCC (46, 47). In contrast to our results in donkeys, Brolund (48) reported that the mean SCC for the Swedish Red and White breed increased from 28 × 10^3^ cells/ml for parity 1 of cows to 72 × 10^3^ cells/ml for parity 4 of cows. The higher lysozyme activity in donkey milk than that reported in raw cow milk might cause the opposite results between donkeys and cows (49). Donkey milk has been reported to have stronger microbial inhibitory activity and to result in lower levels of SCC than the milk of any other species due to its high lysozyme levels (50). However, further investigation is needed to explore the influence of parity on the SCC of donkey milk. Regarding the effect of parity on donkey milk yield, parity 3 donkeys produced more milk than parity 1 and 2 donkeys. Donkey milk productivity is usually influenced by the foaling season, heat stress, milking regime, age, and lactation stage (51). Bordonaro et al. (43) demonstrated that donkey milk production can be influenced by age, with younger donkeys (6–10 years) producing 13% more milk than older donkeys (>10 years). There was some confounding between parity and age in this survey, the donkeys were in the range of 3–12 years old. Further research is necessary to separate the effects of parity and age on the milk yield of donkeys.

### 4.6. Future prospective of donkey industry in China

With industrialization and mechanization of agriculture, the donkey stock and donkey pure breeds has declined dramatically (4). However, the genetic resources of domesticated donkey breeds are non-renewable wealth of human beings and could provide abundant breeding materials for donkey breeding and genetic improvement (52). The indigenous donkey breeds are the result of long-term ecological conditions, genetic drift and natural selection (4). Therefore, there is an urgent need to characterize and protect the genetic diversities of local donkey breeds. The current nationwide survey on the situation of domestic donkeys under the large-scale donkey farming systems provided foundational reference for characterizing the genetic diversities and donkey performance. The donkey original breeding farms in China are crucial for the protection of local donkey breed resources. Nowadays, Chinese citizens appear to be more receptive to the importance of environmental and biodiversity preservation, with the possibility of rediscovering the role of donkeys in a perspective of more sustainable agriculture (4).

In addition, compared to the extensive conditions on smallholder farms (16), the large-scale donkey farming systems significantly improved the donkey productivity and promoted local farmers' income, sustainable utilization of resources, ecological and cultural heritage protection. But factors such as donkey health care, management and nutrition during breeding, fattening and lactation that influence the milk and meat performance of donkeys have not been investigated in detail. We may get some insights from China dairy cattle industry concerning the modern production technology and feed management techniques in order to improve production efficiency and product quality of donkeys. The government should continue to support farmers and farmer cooperatives to building or upgrading more intensive donkey farms and donkey processing facilities. Furthermore, the innovation and full exploitation of donkey products (meat, milk) and byproducts (heads, skins, bones, and viscera) in China has not been well studied (4). The information regarding the qualitative and quantitative characterization of the nutritional and functional components of donkey products also need to be collected in the future to improve the competitiveness and sustainability of the donkey industry.

## 5. Conclusion

The present study is the first comprehensive survey of donkey original breeding farms using questionnaires online to investigate the status of a Chinese donkey population. Nowadays, China has developed a donkey reserve system based on national, provincial and non-governmental (self-own) donkey original breeding farms. These large-scale donkey breeding farms were mainly concentrated in Northern of China, and 52% of them keep their donkeys with a stocking density of 100–500 donkeys. Sixteen local donkey breeds including large-sized, medium-sized and small-sized breeds were collected in our survey, and Dezhou donkey are currently prevalent in China with a percentage of more than 57% of the total donkeys. The parameters related donkey reproduction and donkey productivity (growth and lactation performance) were different across national, provincial and self-own donkey original breeding farms. The artificial insemination has been performed in donkey original breeding farms with an average proportion of 73%. In addition, our results indicate that the donkey breeds with different body size may also affect the donkey reproduction parameters and productivity, with the large-sized donkeys had better performance compared to the small-sized donkeys. However, issues such as donkey health care, management and nutrition during breeding, fattening and lactation that influence donkey productivity under large-scale farm systems need further investigation in the future.

## Data availability statement

The original contributions presented in the study are included in the article/Supplementary material, further inquiries can be directed to the corresponding author.

## Ethics statement

The animal study was reviewed and approved by Animal Welfare Committee of Liaocheng University.

## Author contributions

ZZ contributed to the manuscript writing, editing, data generation and analysis, revision, and the general ideas of the manuscript. ZZ, BH, YW, MZ, and GL contributed to all the sample collection, results discussion, and collection of literature. CW contributed to the conceptualization of the study, study fund support, results discussion, and draft revision. All authors read and approved the manuscript.
