# Supplementary material for: A survey report on the donkey original breeding farms in China: Current aspects and future prospective
Source: Front Vet Sci. 2023 Mar 16;10:1126138. doi: 10.3389/fvets.2023.1126138 (PMC10060844; doi:10.3389/fvets.2023.1126138)
Supplement: Supplementary file 1 [file Data_Sheet_1.docx]

Supplementary Material

# Supplementary Figures

**Figure S1.** The number of donkey herds from 2011 to 2021

| 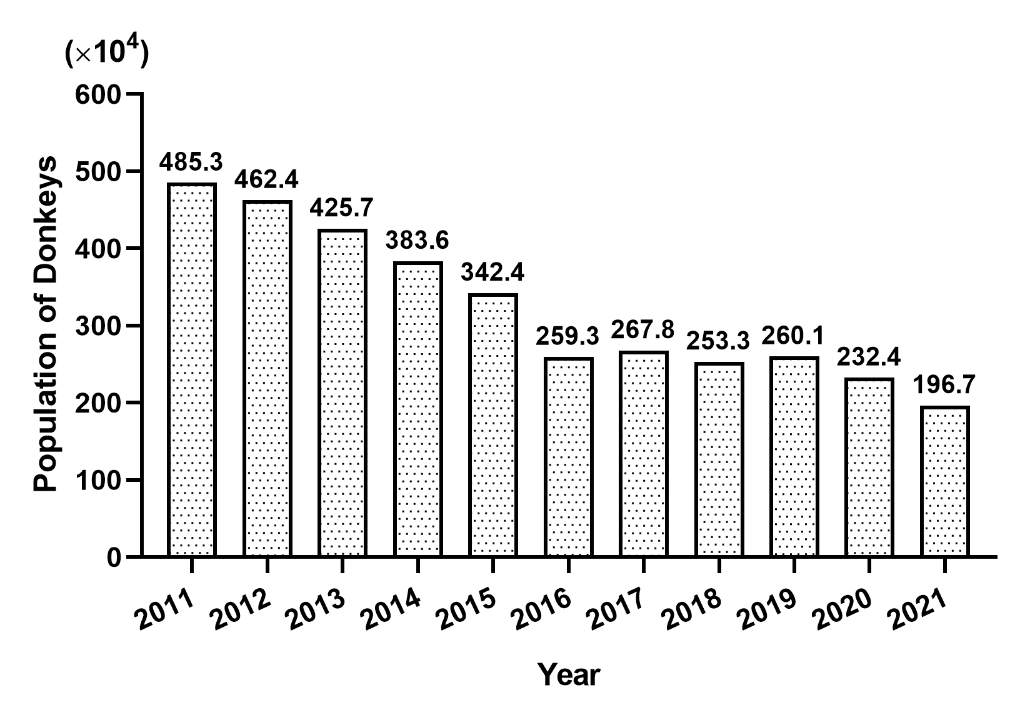 |
| --- |

**Figure S2.** Total numbers of donkeys and the donkey herd composition

| 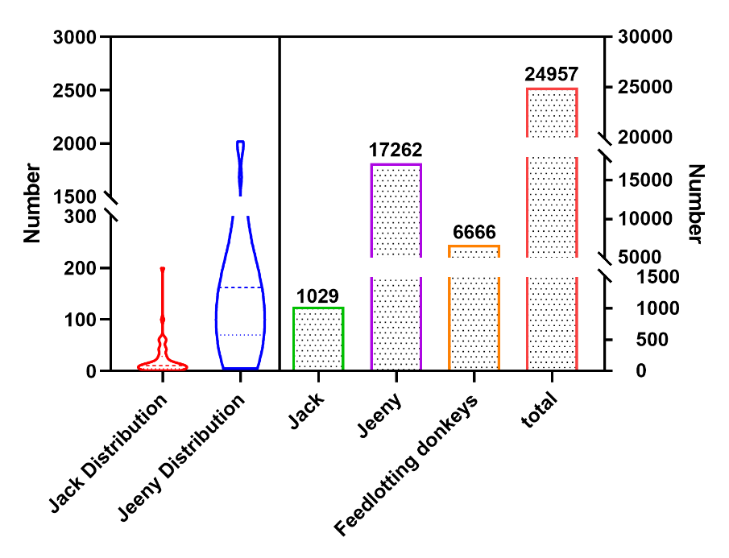 |
| --- |

# Supplementary Tables

# Table S1: Questionnaires for Donkey Original Breeding Farms

| **Basic information of donkey original breeding farms** | | | | | | | | | | | | | | | | | | | | |
| --- | --- | --- | --- | --- | --- | --- | --- | --- | --- | --- | --- | --- | --- | --- | --- | --- | --- | --- | --- | --- |
| Farm name | | | | | | | | | |  | | | | | | | | | | |
| Address | | | | | | | | | |  | | | | | | | | | | |
| Managor (voluntary) | | | | | |  | | | | Email | | | | | |  | | | | |
| National | |  | | | | | Provincial | | |  | | | | Self-Own | | | | |  | |
| Total Employees | | | | | | | | | |  | | | | | | | | | | |
| University-educated | | | | | |  | | | | College-educated | | | | | |  | | | | |
| High-school graduates | | | | | |  | | | | Others | | | | | |  | | | | |
| Donkey breed | | | |  | | | | | | Number of Jeenies | | | | | |  | | | | |
| Livestock on hand | | | |  | | | | | | Number of Jacks | | | | | |  | | | | |
| **Reproductive parameters of jacks** | | | | | | | | | | | | | | | | | | | | |
| Total numbers (n) | | | | | | | | | | | | | | |  | | | | | |
| Age of first semen collection (year) | | | | | | | | | | | | | | |  | | | | | |
| Sperm production (mL/day) | | | | | | | | | | | | | | |  | | | | | |
| Sperm motility (%) | | | | | | | | | | | | | | |  | | | | | |
| The proportion of artificial insemination (%) | | | | | | | | | | | | | | |  | | | | | |
| **Reproductive parameters of jennies** | | | | | | | | | | | | | | | | | | | | |
| Total numbers (n) | | | | | | | | | | | | | | |  | | | | | |
| Age at first foaling (month) | | | | | | | | | | | | | | |  | | | | | |
| Foaling Interval (day) | | | | | | | | | | | | | | |  | | | | | |
| Foaling alive rate (%) | | | | | | | | | | | | | | |  | | | | | |
| Weaning age (month) | | | | | | | | | | | | | | |  | | | | | |
| Proportion of artificial insemination (%) | | | | | | | | | | | | | | |  | | | | | |
| **Growth performance of donkeys** | | | | | | | | | | | | | | | | | | | | |
| Age | | | Gender | | | Numbers (n) | | Body weight (kg) | | | Body height (cm) | | Body length (cm) | | | | Thoracic girth  (cm) | | | Cannon bone girth (cm) |
| Birth | | | Male | | |  | |  | | |  | |  | | | |  | | |  |
|  |  |  | Female | | |  | |  | | |  | |  | | | |  | | |  |
| 3-month-old | | | Male | | |  | |  | | |  | |  | | | |  | | |  |
|  |  |  | Female | | |  | |  | | |  | |  | | | |  | | |  |
| 6-month-old | | | Male | | |  | |  | | |  | |  | | | |  | | |  |
|  |  |  | Female | | |  | |  | | |  | |  | | | |  | | |  |
| 12-month-old | | | Male | | |  | |  | | |  | |  | | | |  | | |  |
|  |  |  | Female | | |  | |  | | |  | |  | | | |  | | |  |
| 18-month-old | | | Male | | |  | |  | | |  | |  | | | |  | | |  |
|  |  |  | Female | | |  | |  | | |  | |  | | | |  | | |  |
| 24-month-old | | | Male | | |  | |  | | |  | |  | | | |  | | |  |
|  |  |  | Female | | |  | |  | | |  | |  | | | |  | | |  |
| **Lactation performance of dairy donkeys** | | | | | | | | | | | | | | | | | | | | |
| Parity | Total number (n) | | | | Milk production (kg) | | | | Fat content  (g/ 100mL) | | | Protein content (g/ 100mL) | | | | | | SCC  (×10^3^ cells/mL) | | |
| 1 |  | | | |  | | | |  | | |  | | | | | |  | | |
| 2 |  | | | |  | | | |  | | |  | | | | | |  | | |
| >3 |  | | | |  | | | |  | | |  | | | | | |  | | |

**
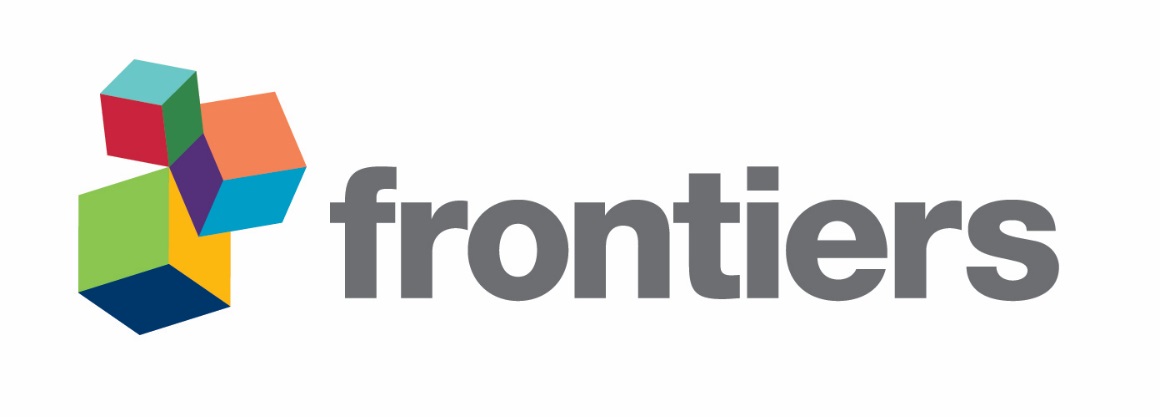
**
